# Transgenic expression of human cytokines in immunodeficient mice does not facilitate myeloid expansion of *BCR-ABL1* transduced human cord blood cells

**DOI:** 10.1371/journal.pone.0186035

**Published:** 2017-10-12

**Authors:** Maria Askmyr, Sofia von Palffy, Nils Hansen, Niklas Landberg, Carl Högberg, Marianne Rissler, Helena Ågerstam, Thoas Fioretos

**Affiliations:** 1 Department of Clinical Genetics, Lund University, Lund, Sweden; 2 Department of Clinical Genetics, University and Regional Laboratories, Region Skåne, Lund, Sweden; European Institute of Oncology, ITALY

## Abstract

Several attempts have been made to model chronic myeloid leukemia (CML) in a xenograft setting but expansion of human myeloid cells in immunodeficient mice has proven difficult to achieve. Lack of cross-reacting cytokines in the microenvironment of the mice has been proposed as a potential reason. In this study we have used NOD/SCID IL2–receptor gamma deficient mice expressing human *SCF*, *IL-3* and *GM-CSF* (NSGS mice), that should be superior in supporting human, and particularly, myeloid cell engraftment, to expand *BCR-ABL1* expressing human cells in order to model CML. NSGS mice transplanted with *BCR-ABL1* expressing cells became anemic and had to be sacrificed due to illness, however, this was not accompanied by an expansion of human myeloid cells but rather we observed a massive expansion of human T-cells and macrophages/histiocytes. Importantly, control human cells without *BCR-ABL1* expression elicited a similar reaction, although with a slight delay of disease induction, suggesting that while *BCR-ABL1* contributes to the inflammatory reaction, the presence of normal human hematopoietic cells is detrimental for NSGS mice.

## Introduction

*In vitro* models of chronic myeloid leukemia (CML) have played a critical role for the understanding of the disease and the development of the tyrosine kinase inhibitor treatment. However, to get a better understanding of cellular interactions, disease pathogenesis and to test novel therapies, *in vivo* models are necessary. In order to create a humanized model of CML, several attempts have been made to transplant primary samples from CML patients or human cord blood (CB) cells transduced with a viral vector expressing the *BCR-ABL1* fusion gene [1–5]. Primary CML patient cells have high interpatient variability in terms of leukemic stem cell frequency and engraftment potential thus making it an unpredictable *in vivo* modelling system [6]. Retroviral transduction of human cells engrafted in immune compromised mice have the potential advantage of being a reproducible model [7]. However, so far, successful *in vivo* modeling of CML has only been achieved in syngenic mouse models [8–11]. Myeloid cell expansion is the key feature in human CML but in humanized mice, this expansion of human *BCR-ABL1* expressing myeloid cells has been difficult to achieve. Other aspects of human CML, like the arrest of B cell development at the pre-B cell stage, have been successfully modeled [3]. Various strains of immunodeficient mice have been tested (non-obese diabetic/severe combined immunodeficient (NOD/SCID), NOD/SCID-β_2_M or NOD/SCID IL2–receptor gamma deficient (NSG) mice) to increase the level of myeloid engraftment, but with limited success. More mouse variants are being developed and one of the more interesting strains for this purpose is a NSG mouse strain that expresses the human cytokines SCF, IL-3 and GM-CSF. These mice, termed NSGS mice, have proven superior to NSG mice in short term engraftment of normal human myeloid cells and also for engraftment of human malignant hematopoietic cells [12–20].

In this study, we investigated whether transplantation of human CB cells transduced with a retroviral vector expressing *BCR-ABL1* would give rise to a CML-like disease in NSGS mice.

## Materials and methods

### Isolation, transduction and transplantation of CD34^+^ cells from CB

Isolation and transduction of cord blood CD34^+^ cells was done as previously described and was approved by the Lund/Malmö Ethical Committee and performed after informed consent in accordance with the Declaration of Helsinki [3, 7]. All animal experiments were approved by The Swedish Board of Agriculture, Malmö/Lund animal ethics committee in Lund, Sweden. For the xenotransplantation assay we used 8–12 week old male or female NSGS mice that were subjected to 200 cGy total body irradiation 18–20 hours prior to transplantation. Mice were given antibiotics (ciprofloxacin) in drinking water and powder food. Mice were transplanted via the tail vein with 3x10^5^ unsorted cells/mouse 24 hours post transduction (initial transduction efficiencies ranging from 2–24%). Counting of white blood cells, red blood cell and platelets in peripheral blood (PB) was performed on an ABX Micros 60 cell counter (Horiba ABX Corporate, Edison, NJ, USA). Mice were monitored daily and sacrificed at signs of illness (anemia and reduced motility).

### Histopathology

Long bones and spleens were collected for histopathology analysis as previously described [3]. Bone and spleen sections were stained using CD68 (Dako, Glostrup, Denmark).

### Flow cytometric analysis

Cells were prepared for flow cytometry as previously described [3] and the following antibodies were used: CD45-APC, CD15-BV605, CD33-PECy7 and Glycophorin A-PE (BD Biosciences, San Jose, CA, USA), CD33-BV421, CD19-PerCPCy5.5, CD14-BV605, CD117-PerCPCy5.5, CD3-PECy7 and FceRI-PE (BioLegend, San Diego, CA, USA).

### Statistics

GraphPad Prism Version 6.0a was used to perform Student’s t-test (unpaired, two-tailed) and log-rank (Mantle-Cox) test was used for survival differences. Mean values are accompanied by standard deviation (SD). P-values ≤ 0.05 were considered statistically significant. Only significant p-values are displayed in the figures.

## Results and discussion

**Fig 1:**
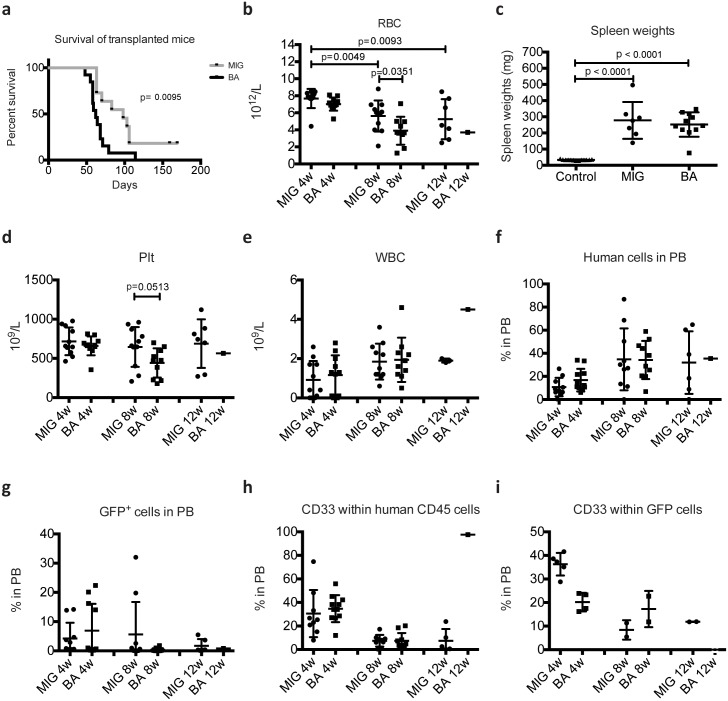
Survival and analysis of human cell engraftment and lineage distribution in PB of transplanted NSGS mice. NSGS mice transplanted with control cells (MIG mice) and *BCR-ABL1* expressing cells (BA mice) were sacrificed at signs of illness and survival time is shown in (A). Red blood cells (RBC) measured in PB is shown in (B). The spleen weights of mice at time of sacrificed is shown in (C). Untreated NSGS mice 12–15 weeks of age are used as control for spleen weights. Platelet (Plt) counts (D), white blood cell (WBC) count (E), level of human CD45^+^GFP^-^ (F) and CD45^+^GFP^+^ (G) and the level of human CD33^+^ myeloid cells (H and I) was measured in PB at 4, 8 and 12 weeks post transplantation. GraphPad Prism Version 6.0a was used to perform Student’s t-test (unpaired, two-tailed) for statistical analysis and variation is shown as standard deviation. P-values ≤ 0.05 were considered statistically significant. Log-rank (Mantle-Cox) test was used for survival differences.

In an attempt to model human CML, NSGS mice were transplanted with human CD34^**+**^ stem/progenitor cells expressing either *BCR-ABL1* together with green fluorescent protein (GFP) (BA mice) or control cells expressing only GFP (MIG mice). As shown in Fig 1A, BA mice succumbed to disease, starting 50 days post transplantation. However, also MIG mice (7 out of 9) fell ill and had to be sacrificed, although with a slight delay of approximately 2–3 weeks (Fig 1A). This is in sharp contrast to our previous findings using NSG mice where only BA mice became ill [3]. All transplanted mice developed progressive anemia as measured by red blood cell numbers (RBC) in PB (Fig 1B) and at the time of sacrifice, spleen weights were increased in both BA and MIG mice (Fig 1C). Platelets were only reduced in BA mice and this reduction was evident at 8 weeks post transplantation (Fig 1D). In our previous studies using NSG mice, transplantation of *BCR-ABL1* transduced CD34^+^ CB cells did not result in elevated PB white blood cell count (WBC) [3], a feature that is characteristic in CML patients. We analyzed the WBC in PB of the transplanted NSGS mice but could not detect any increase (Fig 1E), suggesting that *BCR-ABL1* does not induce a massive expansion of human myeloid cells.

Although we could not detect higher peripheral levels of WBC, *BCR-ABL1* could potentially induce lineage skewing, preferentially expanding myeloid cells. To evaluate this, we performed flow cytometric analysis of PB. As shown in Fig 1F, the level of human cell engraftment was similar between BA and MIG mice. The level of GFP^+^ cells was in general low and did not differ between BA and MIG mice (Fig 1G). We then used the myeloid marker CD33 but could not detect any differences within either CD45^+^GFP^-^ (Fig 1H) or CD45^+^GFP^+^ cells (Fig 1I) between the groups. Thus, although mice succumb to anemia, characteristic symptoms of CML such as expansion of myeloid cells in the periphery were not observed. Unexpectedly, also the mice transplanted with control cells not expressing *BCR-ABL1* became ill, suggesting that the presence of normal human cells induced detrimental effects.

**Fig 2:**
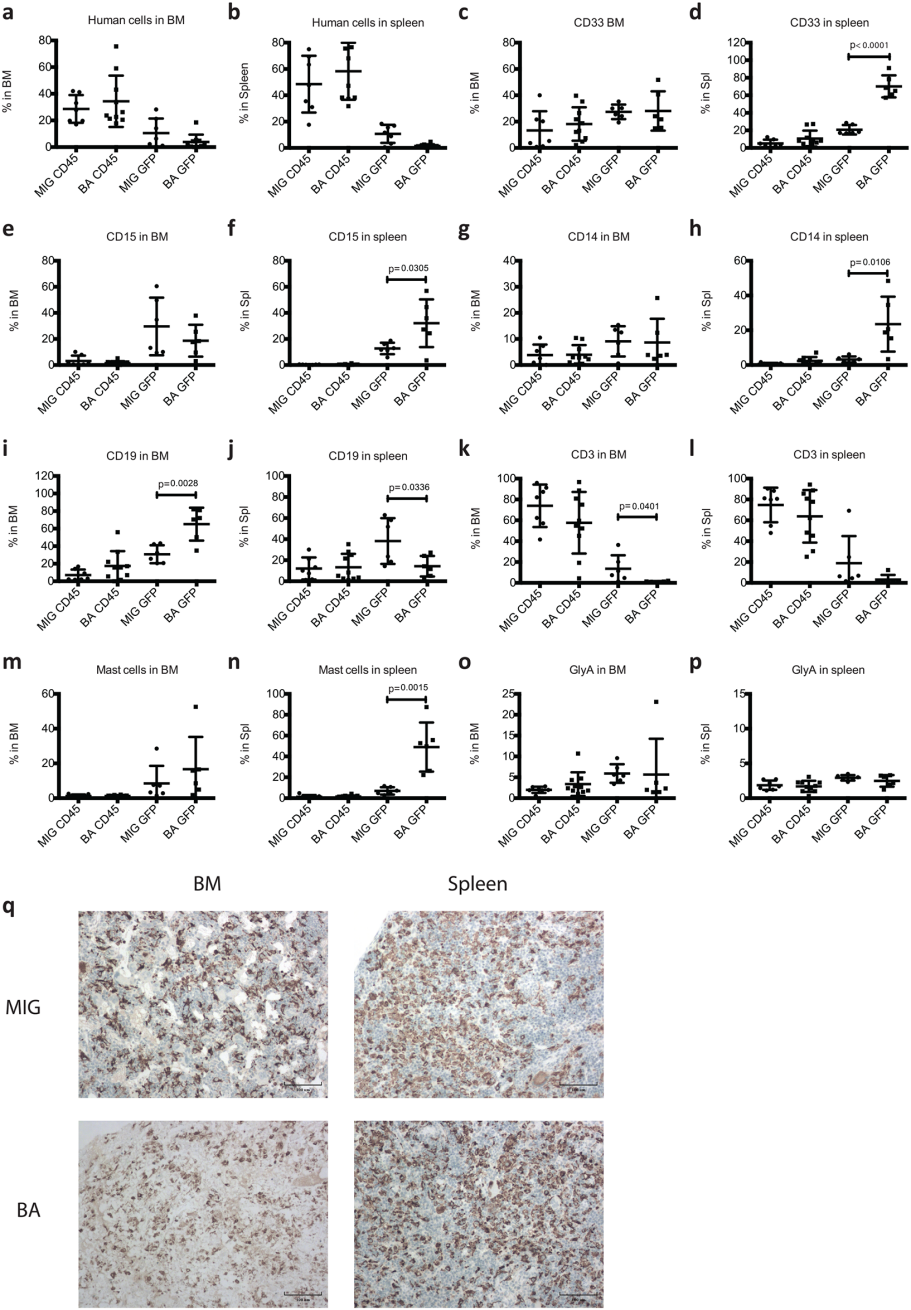
Analysis of level and lineage distribution of human cell engraftment in BM and spleen of transplanted NSGS mice. NSGS mice transplanted with control cells (MIG mice) and *BCR-ABL1* expressing cells (BA mice) were sacrificed when signs of illness appeared. BM and spleen cells were analyzed using flow cytometry and the level of human cell engraftment in BM (a) and spleen (b) and the lineage distribution (c-p) are shown. Paraffin sections of BM and spleen were stained for CD68 showing the presence of macrophages/histiocytes. Scale bar 100 μm (q). GraphPad Prism Version 6.0a was used to perform Student’s t-test (unpaired, two-tailed) for statistical analysis and variation is shown as standard deviation. P-values ≤ 0.05 were considered statistically significant.

We next analyzed the level and lineage distribution of human cells within bone marrow (BM) and spleen (Fig 2A–2P). Human cell engraftment was comparable between the groups (Fig 2A and 2B). Interestingly, although 300 000 cells/mouse were transplanted compared to 200 000 cells/mouse in our previously study using NSG mice [3], human cell engraftment was lower in the NSGS mice. Analysis of BM showed that GFP frequency was low, particularly in the BA mice (Fig 2A) and the only significant lineage differences among the engrafted human cells between BA and MIG mice were an increase in B-cells and decreased T-cells in BA mice (Fig 2I and 2K). In the spleen, the differences between the BA and MIG GFP^+^ populations were more prominent. The myeloid CD33^+^ population was increased in BA mice (Fig 2D) and the lymphoid populations were low (Fig 2J and 2L). Again, as observed in our NSG mice, mast cells were expanded among the *BCR-ABL1* expressing cells in the spleen (Fig 2N) [3]. The most prominent features in both BA and MIG mice were however, the massive expansion of human T-cells (Fig 2K and 2L) and human macrophages/histiocytes (Fig 2Q) in both BM and spleen. This suggests that the presence of human cells, not only the *BCR-ABL1* transduced cells, elicits an inflammatory reaction in NSGS mice.

**Fig 3:**
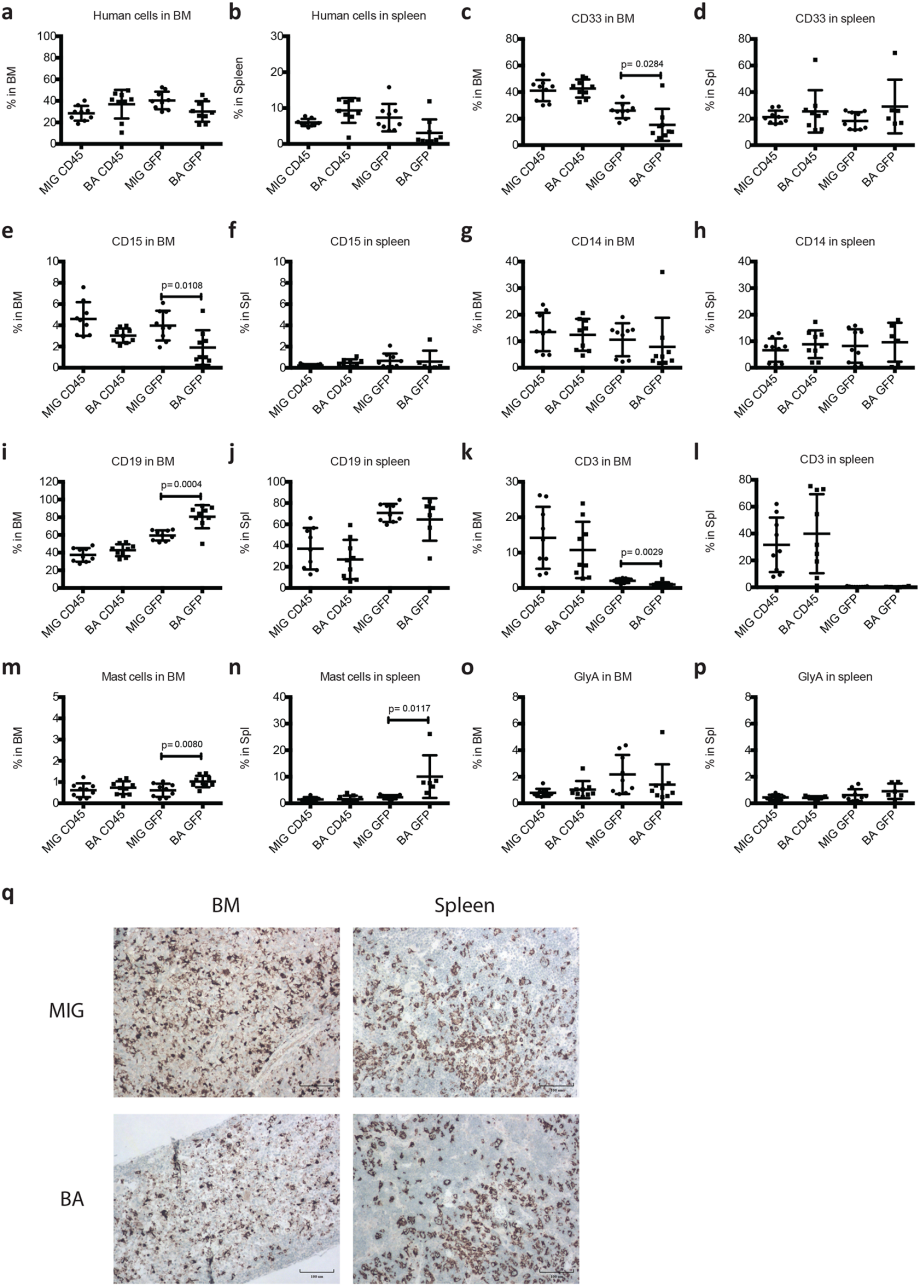
Analysis of level and lineage distribution of human cell engraftment in NSGS mice 5 weeks post transplantation. NSGS mice transplanted with control cells (MIG mice) and *BCR-ABL1* expressing cells (BA mice) were sacrificed 5 weeks post transplantation. BM and spleen cells were analyzed using flow cytometry and the level of human cell engraftment in BM (a) and spleen (b) and the lineage distribution (c-p) are shown. Paraffin sections of BM and spleen were stained for CD68 showing the presence of macrophages/histiocytes. Scale bars 100 μm (q). GraphPad Prism Version 6.0a was used to perform Student’s t-test (unpaired, two-tailed) for statistical analysis and variation is shown as standard deviation. P-values ≤ 0.05 were considered statistically significant.

Myeloid expansion induced by *BCR-ABL1* in NSGS mice might be masked by the expansion of T-cells and macrophages/histiocytes. Thus, transplanted mice were sacrificed 5 weeks post transplantation before the onset of illness but long enough after transplantation to allow for human cell expansion. At this time point, the level of GFP^+^ cells in the BM (Fig 3A) but not in the spleen (Fig 3B) was higher compared to mice that were analyzed when illness appeared. However, we did not observe an increase of myeloid (Fig 3C, 3E and 3G) or erythroid cells (Fig 3O). Only a slight increase of mast cells (Fig 3M) and B-lymphocytes (Fig 3I and 3K) was observed in the BM of BA mice. The only change to lineage distribution in the spleen was an increase of *BCR-ABL1*^+^ mast cells (Fig 3D, 3F, 3H, 3J, 3L, 3N and 3P). Again, although not as prominent as at time of illness, human T-cells (Fig 3K and 3L) and macrophages/histiocytes (Fig 3Q) were expanded. In conclusion, although a significant increase of *BCR-ABL1*^+^ myeloid cell populations were seen in the spleens of mice sacrificed when sick (Fig 2), these mice had low engraftment of transduced cells (mean 1,5% compared to 10,73% in MIG control mice, Fig 2B) making these results difficult to interpret. When mice were sacrificed after 5 weeks, before the onset of disease (Fig 3), engraftment numbers of transduced human cells in the spleen were more similar between the groups (3,1% in BA mice vs 7,3% in MIG mice). At this stage, these differences in myeloid cells were no longer present.

Collectively, our results confirm previous studies where myeloid cell expansion using *BCR-ABL1* overexpression in xenograft settings has proven to be difficult [3–5]. Here we used one of the most permissive mouse strains available when it comes to human myeloid engraftment. However, a CML-like disease was not observed. Unexpectedly, also normal human hematopoiesis induced anemia and an inflammatory reaction. However, a recent report describes development of progressive anemia and an inflammation like phenotype in NSGS mice after transplantation of unfractionated CB cells. This was not the result of human lymphoid cells since removal of lymphoid cells by the use of lymphocyte suppressive therapies such as steroids or antibody-mediated ablation of B and T cells did not reverse the symptoms. Only by eliminating the entire human graft or the human myeloid cells (by an anti-CD33 antibody) could the disease be reversed [21]. This supports our finding that unmodified human myeloid cells are detrimental for the NSGS mice and that it is unlikely that treatment with antibodies targeting B and T cells would inhibit the development of the inflammatory disease that we observe in our mice.

The difficulty in engrafting and expanding human CML cells in mice indicates that although the murine microenvironment seem to be supportive of human leukemia stem cells, it is lacking important regulators for CML stem cells [22]. One way of addressing this is to establish a human microenvironment in immunodeficient mice. This has successfully been done for myeloid dysplastic syndrome (MDS). Both co-transplantation of human mesenchymal cells and introduction of human bone scaffolds coated with human mesenchymal cells allow for engraftment and expansion of human MDS stem cells [23, 24]. Humanized bone scaffolds were recently tested also for CML and transduced human CB CD34^+^ cells expressing *BCR-ABL1* could induce both lymphoid and myeloid leukemia in transplanted NSG mice [25]. Although myeloid leukemia was found in primary mice, secondary transplants resulted only in lymphoid leukemia. This shows however, that the microenvironment is important in the development of human leukemia in mice and further work along this line will be needed to enable studies of novel mechanism regulating CML stem cells, the disease evolution and for evaluation of new therapeutic strategies.

## Supporting information

S1 File NC3Rs ARRIVE guidelines checklist (fillable). (PDF)
